# Physicochemical Properties and Biological Activities of Quinoa Polysaccharides

**DOI:** 10.3390/molecules29071576

**Published:** 2024-04-01

**Authors:** Xucheng Zhu, Guiyan Yang, Yingbin Shen, Liqiong Niu, Yao Peng, Haiting Chen, Haimei Li, Xinquan Yang

**Affiliations:** 1 School of Life Sciences, Guangzhou University, Guangzhou 510006, China; zhuxucheng@e.gzhu.edu.cn (X.Z.); shenybin412@gmail.com (Y.S.); niuliqiong@163.com (L.N.); pengyao_79@163.com (Y.P.); 1914400047@e.gzhu.edu.cn (H.C.); 2 College of Veterinary Medicine, China Agricultural University, Beijing 100193, China; yangguiyan1990@hotmail.com

**Keywords:** quinoa polysaccharides, chemical composition, antioxidant activity, anti-proliferation activity, anti-microbial activity

## Abstract

Quinoa, known as the “golden grain” for its high nutritional value, has polysaccharides as one of its sources of important nutrients. However, the biological functions of quinoa polysaccharides remain understudied. In this study, two crude polysaccharide extracts of quinoa (Q-40 and Q-60) were obtained through sequential precipitation with 40% and 60% ethanol, with purities of 58.29% (HPLC) and 62.15% (HPLC) and a protein content of 8.27% and 9.60%, respectively. Monosaccharide analysis revealed that Q-40 contained glucose (Glc), galacturonic acid (GalA), and arabinose (Ara) in a molar ratio of 0.967:0.027:0.006. Q-60 was composed of xylose (xyl), arabinose (Ara), galactose, and galacturonic acid (GalA) with a molar ratio of 0.889:0.036:0.034:0.020. The average molecular weight of Q-40 ranged from 47,484 to 626,488 Da, while Q-60 showed a range of 10,025 to 47,990 Da. Rheological experiments showed that Q-40 exhibited higher viscosity, while Q-60 demonstrated more elastic properties. Remarkably, Q-60 showed potent antioxidant abilities, with scavenging rates of 98.49% for DPPH and 57.5% for ABTS. Antibacterial experiments using the microdilution method revealed that Q-40 inhibited the growth of methicillin-resistant Staphylococcus aureus (*MRSA*) and *Escherichia coli* (*E. coli*), while Q-60 specifically inhibited MRSA. At lower concentrations, both polysaccharides inhibited MDA (MD Anderson Cancer Center) cell proliferation, but at higher concentrations, they promoted proliferation. Similar proliferation-promoting effects were observed in HepG2 cells. The research provides important information in the application of quinoa in the food and functional food industries.

## 1. Introduction

*Chenopodium quinoa* Willd, an annual herb originating in South America, is now widely grown in Asia, Africa, and Europe [1,2]. Quinoa cultivation began in Tibet in 1988 and was later introduced to other regions such as Gansu, Shanxi, Qinghai, Hebei, and Inner Mongolia [3]. It is highly valued for its rich content of proteins, amino acids, starch, cellulose, minerals, and other beneficial nutrients, which impart various health benefits including anti-inflammatory, antibacterial, antitumor, and anti-aging effects [4,5,6]. Recognized by the Food and Agriculture Organization of the United Nations (FAO) as the only whole-nutrient plant for humans, quinoa’s nutritional profile meets the dietary needs of people. Globally, quinoa is cultivated in an area of 188,878 ha, producing 175,188 tons [7]. Due to its nutritional value, quinoa is often referred to as “the super grain” and “the mother of grain,” while in China, most of the grain produced is used for storage [8].

With increasing cultivation globally, quinoa is poised to become an essential food crop and industrial raw material in the 21st century. In developing countries, where balanced diets with adequate and high-quality protein are challenging to achieve, quinoa holds the potential to address this nutritional gap [9]. Conversely, in developed countries, quinoa can be integrated into snacks, bread, pasta, and prepared foods to enhance their nutritional quality [9]. Investigating the nutritional composition of Chinese quinoa will further improve our understanding and utilization of different quinoa varieties [10]. The consumption of quinoa is being encouraged, as several recent studies have indicated its potential in reducing the risk of cardiovascular disease [11].

Polysaccharides extracted from quinoa, known as biological response modifiers (BRM), play a vital role in the growth and development of organisms [12,13,14]. Generally, they are composed of glucose, rhamnose, arabinose and galactose [15]. These polysaccharides have diverse biological functions, including energy storage, structural support, and physical defense, as well as critical physiological functions like antioxidant [16,17], hypolipidemic [18,19], hypoglycemic [20], anti-tumor [21], and immune-enhancing effects [22,23].

While the extraction of polysaccharides from various plants has been extensively studied, research on quinoa polysaccharides remains limited. In this study, we prepared crude quinoa polysaccharides through hot water extraction and subsequently separated them into different fractions using graded ethanol precipitation. We investigated the yield, polysaccharide content, rheological characteristics, protein content, and antioxidant activity of each fraction. Additionally, we analyzed the monosaccharide composition, molecular weight, and thermogravimetric properties of Q-40 and Q-60, two specific quinoa polysaccharides. Furthermore, we evaluated their in vitro anti-proliferative activity and antibacterial abilities. This study contributes valuable information for future structural identification and product development involving quinoa polysaccharides.

## 2. Results

### 2.1. The Yield of Crude Polysaccharides and Its Component Analysis

Ethanol can reduce the dielectric constant of polysaccharide solutions, thereby decreasing the interaction of polysaccharides with water and leading to the precipitation of polysaccharide polymers. In this study, we investigated the impact of 40% and 60% concentrations of ethanol on the precipitation and fractionation of quinoa polysaccharides. Two quinoa polysaccharides, namely Q-40 and Q-60, were obtained through ethanol fractionation. After freeze-drying, Q-40 appeared as a light yellow flocculent substance, while Q-60 exhibited a darker color compared to Q-40. According to the results of the phenol–sulfuric acid experiment and the colloidal Coomassie brilliant blue experiment, the polysaccharide content of Q-40 was found to be 58.29%, with a protein content of 8.27%, whereas Q-60 contained 62.15% polysaccharide and 9.6% protein content.

### 2.2. Rheological Characteristics

**Figure 1:**
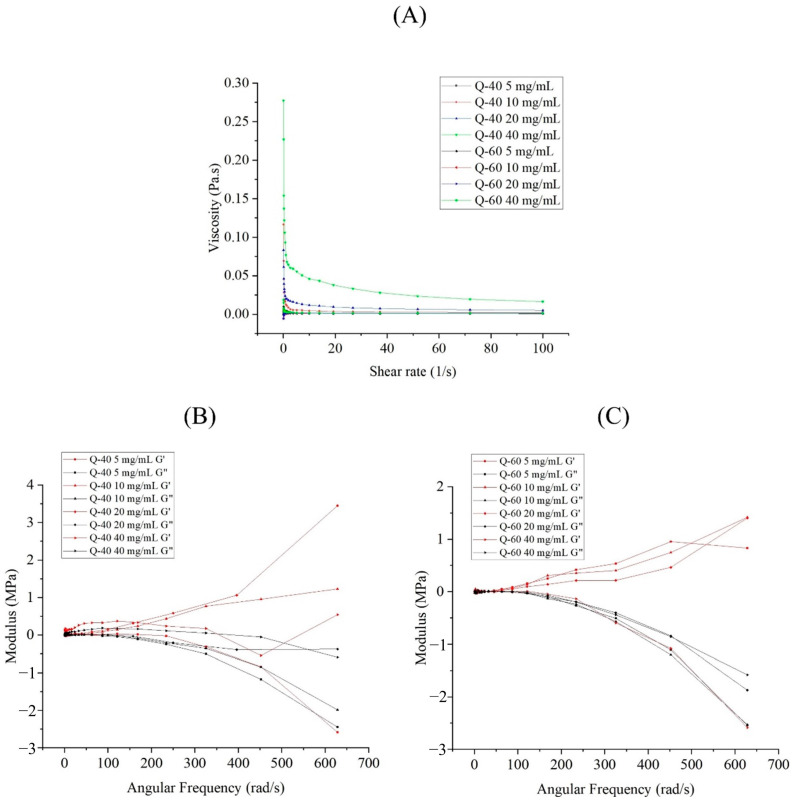
Rheological properties of Q-40 and Q-60. (**A**) Effect of concentration on the viscosity of Q-40 and Q-60 solutions; (**B**) effect of concentration on the storage modulus (G′); (**C**) effect of concentration on the loss modulus (G″) of Q-40 and Q-60 solutions.

The viscosity of Q-40 and Q-60 sample solutions increased as the concentration increased, with Q-60 solutions consistently exhibiting higher viscosity than Q-40 solutions at equivalent concentrations (Figure 1A). Furthermore, all solutions displayed shear-thinning behaviors, indicating their pseudoplastic properties, as the shear rate increased.

Figure 1B,C presented the storage modulus (G′) and loss modulus (G″) of Q-40 (a) and Q-60 (b) against frequency (0.1% strain), providing insights into the viscoelastic properties of the polysaccharides. Both Q-40 and Q-60 solutions displayed frequency and concentration-dependent behavior in terms of their storage and loss moduli. Specifically, the storage modulus (G′) of Q-40 solutions at concentrations of 10 mg/mL and 20 mg/mL was higher than the loss modulus (G″), indicating elastic behavior in those cases. Conversely, at 5 mg/mL and 40 mg/mL concentrations, the G′ and G″ intersected. The 5 mg/mL solution exhibited viscous properties at high frequency (G′ < G″) and elastic properties at low frequency (G′ > G″), while the 40 mg/mL solution’s behavior became unstable as the frequency increased, showing several intersections. This may be attributed to the complex network structure formed by the high concentration of Q-40 polysaccharides.

In contrast, Q-60 solutions showed concentration-dependent viscoelastic properties. At lower concentrations, they displayed elastic behavior (G′ > G″), whereas higher concentrations led to viscous behavior (G′ < G″) as the frequency increased.

The data highlights distinct rheological properties between Q-40 and Q-60, underscoring the significant influence of ethanol concentration on the precipitation of quinoa polysaccharides. Overall, polysaccharides precipitated with 40% ethanol exhibited more viscous behavior, while those extracted with 60% ethanol displayed more elastic components.

### 2.3. Monosaccharide Composition

**Figure 2:**
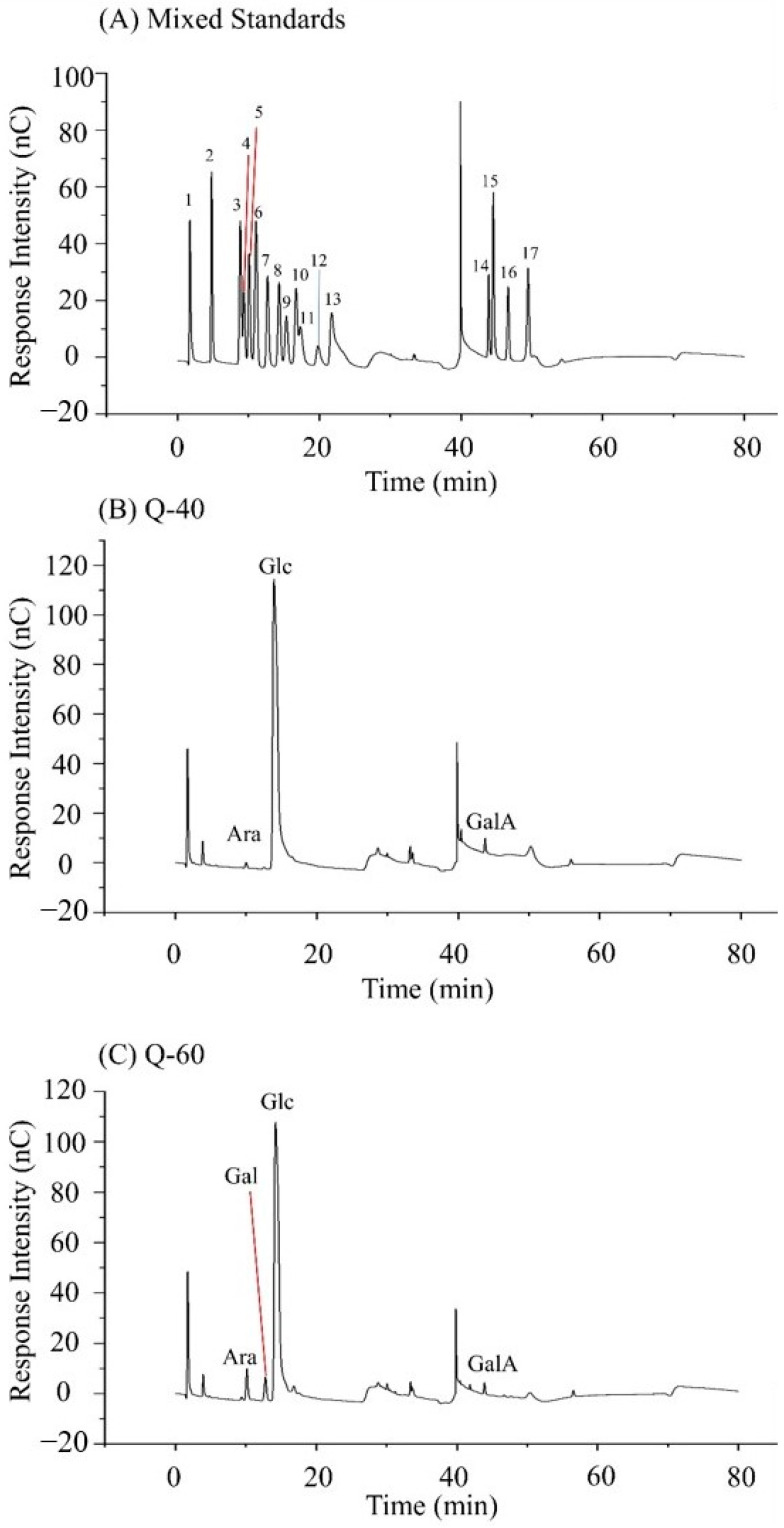
(**A**) is the HPLC chromatogram of the mixture of 16 monosaccharide standards (1. NaOH; 2. Fuc; 3. GalN; 4. Rha; 5. Ara; 6. GlcN; 7. Gal; 8. Glc; 9. GlcNAc; 10. Xyl; 11. Man; 12. Fru; 13. Rib; 14. GulA; 15. ManA; 16. GlaA; 17. GlcA); (**B**) is the HPLC chromatogram of Q-40; and (**C**) is the HPLC chromatogram of Q-60.

Figure 2A displays the HPLC chromatogram of 16 monosaccharide standard derivatives, and standard curves were generated based on the peak times and peak areas of these monosaccharides. Arabinose, glucose, and galacturonic acid were detected in Q-40 with a molar ratio of 0.006:0.967:0.027 (Figure 2B and Table 1). In the case of Q-60, the retention times were recorded as 10.159, 12.784, 14.234, and 43.934 min, indicating the presence of arabinose, galactose, glucose, and galacturonic acid in the sample, with a molar ratio of 0.036:0.034:0.889:0.020, respectively (Figure 2C and Table 1).

It is worth noting that some studies reported quinoa polysaccharides to mainly consist of six sugars: arabinose, rhamnose, xylose, mannose, galactose, and glucose, with glucose being the most abundant among them. The observed differences in monosaccharide composition between Q-60 and the reported findings may be attributed to variations in the raw material origins of quinoa and the methods used for polysaccharide extraction and separation. Such factors can lead to differences in the final polysaccharide composition obtained from different sources of quinoa.

### 2.4. Molecular Weight

**Figure 3:**
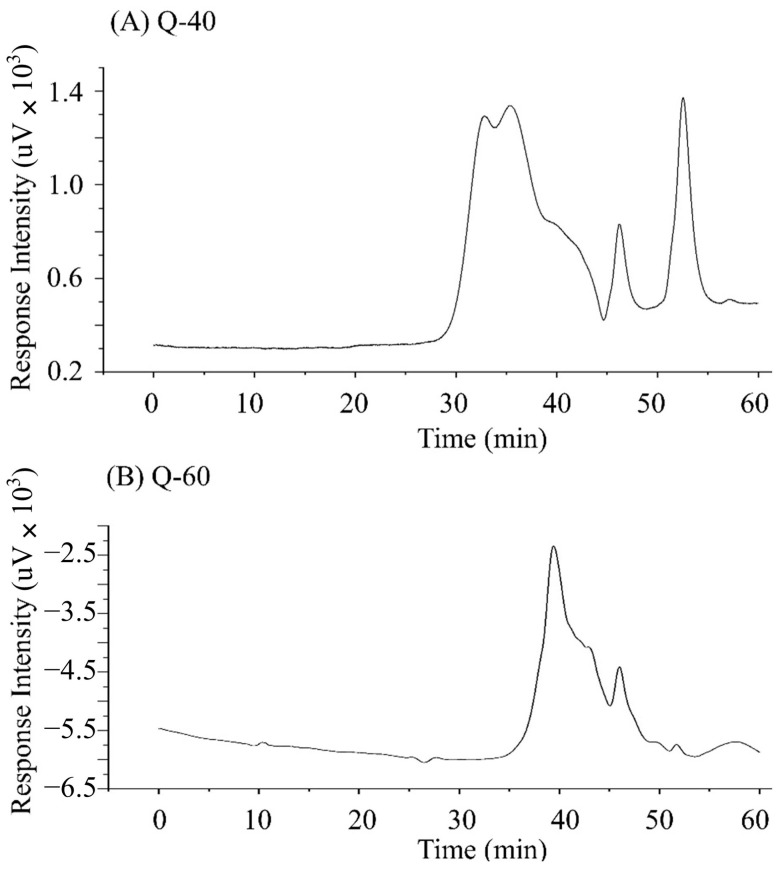
HPGPC diagram of Q-40 and Q-60. (**A**) is the molecular weight distribution of Q-40 and (**B**) is that of Q-60.

Figure 3 presents the molecular weight distribution and purity of quinoa polysaccharides as determined by High-Performance Gel Permeation Chromatography (HPGPC). In the spectrum, Q-40 showed three distinct elution peaks, while Q-60 displayed two. Notably, in both Q-40 and Q-60, peaks subsequent to the salt peaks were observed, hinting at the potential presence of oligosaccharides in the polysaccharide fractions.

The peaks’ asymmetry and varying widths further highlight the polysaccharides’ heterogeneity, suggesting that each sample comprises a blend of multiple polysaccharide entities. This heterogeneity is indicative of the complex nature of the polysaccharide mixtures extracted from quinoa.

Moreover, there was a noticeable difference in the molecular weights of the quinoa polysaccharides extracted using 40% and 60% ethanol, as detailed in Table 2. A trend of decreasing molecular weight with an increase in ethanol concentration was observed. The Molecular Weight Distribution Index (MWDI) revealed that polysaccharides in Q-60 had a relatively narrower distribution than those in Q-40.

### 2.5. SEM (Scanning Electron Microscope) Analysis

Upon observation under a 500× microscope, Q-40 displayed a cluster-turned flake structure with varying sizes. The surface appeared smooth and intact, indicating strong polymerization and interactions between the polysaccharide components. Further examination under a 5000× microscope revealed characteristically large indentations on the polysaccharide surface, accompanied by internal holes.

**Figure 4:**
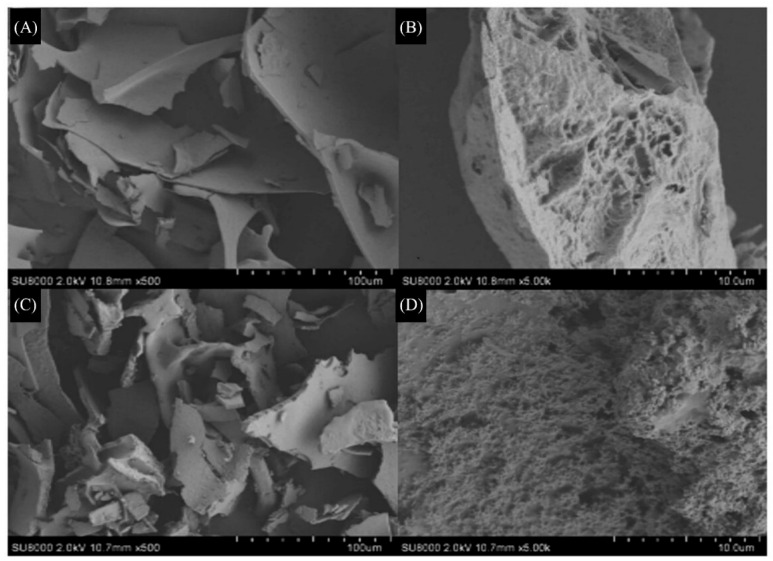
Scanning electron micrographs of quinoa polysaccharides (**A**) Q-40 600×, (**B**) Q-40 6000×; (**C**) Q-60 600×, (**D**) Q-60 6000×.

In contrast, Q-60 exhibited a fragmented polysaccharide structure with diverse sizes and numerous irregular bumps on the surface, forming spherical or ovoid shapes when viewed under a 500× microscope. Upon magnification to 5000×, the surface of the polysaccharides appeared porous, resembling a honeycomb-like structure (Figure 4).

### 2.6. Thermal Analysis

**Figure 5:**
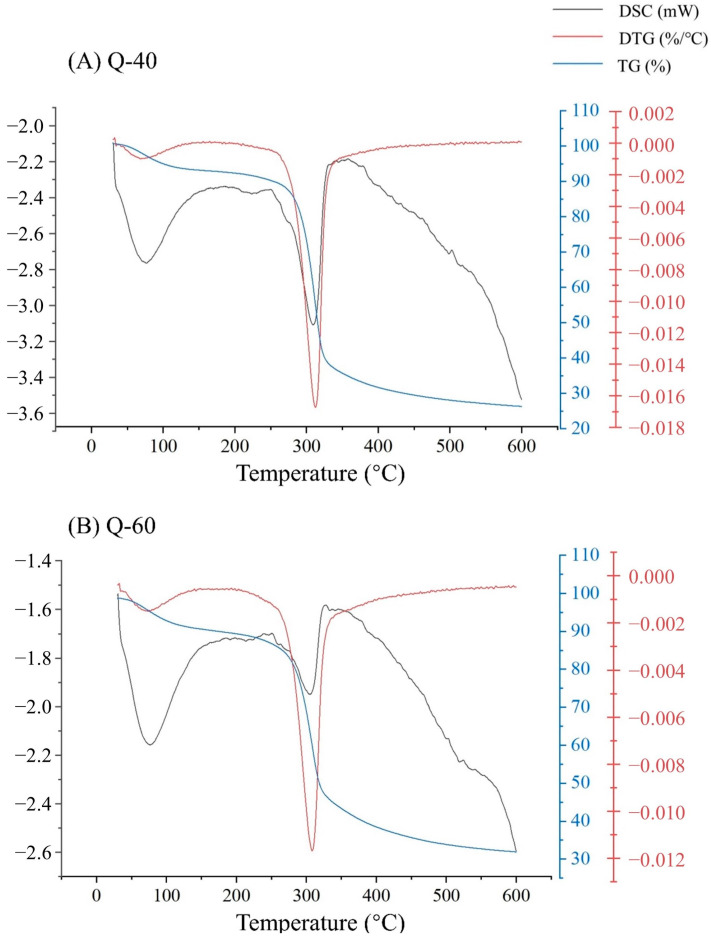
(**A**) DSC, DTG, and TG curves of Q-40; (**B**) DSC, DTG, TG curves of Q-60.

Three primary curves were employed for the thermal analysis, as depicted in Figure 5A,B. The combination of TG (Thermogravimetric Analysis), DTG (Derivative Thermogravimetric Analysis), and DSC (Differential Scanning Calorimetry) curves revealed two distinct thermal weight loss peaks. The initial weight loss peak was observed at approximately 70 °C, with both polysaccharide fractions experiencing a weight loss of around 7%. This weight loss primarily resulted from the evaporation of water present in the samples.

The second weight loss peak occurred within the temperature range of 200 to 510 °C, during which the polysaccharides underwent rapid weight loss. Q-40 lost about 63% of its weight, while Q-60 lost about 56%. This significant weight reduction indicates violent decomposition reactions and oxidation of polysaccharides within this temperature range. However, the decomposition reactions tended to stabilize after reaching 510 °C.

The analysis revealed two main thermal weight loss peaks. The initial peak at approximately 70 °C, where both polysaccharide fractions experienced a 7% weight loss, is indicative of the moisture content. This weight loss is attributed primarily to the evaporation of water present in the polysaccharide samples. The second peak, observed in the range of 200 to 510 °C, marked a phase of rapid weight loss (63% for Q-40 and 56% for Q-60). This substantial reduction in weight is a clear indication of violent decomposition reactions and the oxidation of polysaccharides occurring within this temperature range.

### 2.7. Antioxidant Activity

**Figure 6:**
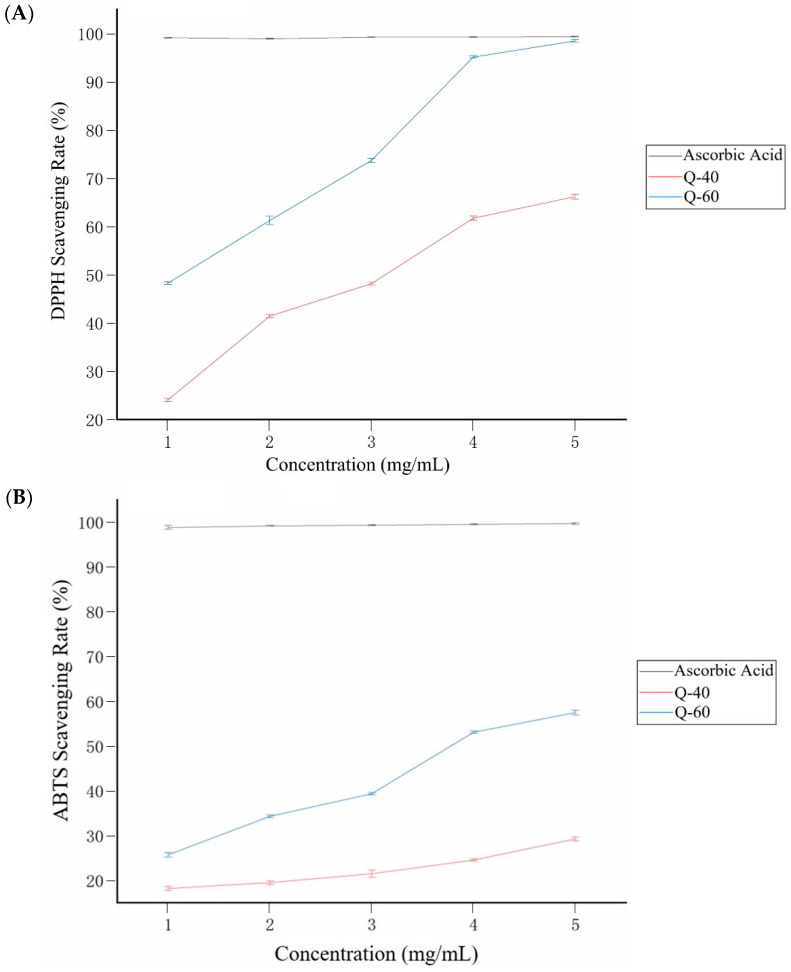
Antioxidant properties of Q-40 and Q-60. (**A**) DPPH radical scavenging ability of ascorbic acid, Q-40, and Q-60; (**B**) ABTS radical scavenging ability of ascorbic acid, Q-40, and Q-60.

Figure 6 demonstrates the scavenging activities of Q-40, Q-60, and ascorbic acid on 1,1-Diphenyl-2-picrylhydrazyl radical 2,2-Diphenyl-1-(2,4,6-trinitrophenyl)hydrazyl (DPPH) and 2,2′-azino-bis(3-ethylbenzothiazoline-6-sulfonic acid (ABTS) radicals. In particular, Q-60 exhibited a significantly higher scavenging rate on DPPH radicals compared to Q-40 (Figure 6A). At a concentration of 5.0 mg/mL, the DPPH radical scavenging ability of Q-40 was measured at 66.18%, whereas Q-60 showed a remarkable DPPH radical scavenging rate of 98.49%. These results indicate that quinoa polysaccharides obtained through ethanol-graded precipitation hold substantial value for effectively scavenging DPPH radicals.

Regarding ABTS radical scavenging (Figure 6B), both Q-40 and Q-60 displayed considerable scavenging abilities; although, their overall effect was weaker than that of ascorbic acid. As the concentration ranged from 0 to 5.0 mg/mL, the ABTS radical scavenging capacity of Q-40 and Q-60 gradually increased. At 5.0 mg/mL, Q-40 demonstrated an ABTS radical scavenging activity of 27.26%, while Q-60 exhibited a higher rate of 57.5%.

The DPPH radical scavenging assay showed that Q-60 has a significantly higher scavenging rate compared to Q-40. This indicates that the ethanol-graded precipitation method used to obtain Q-60 polysaccharides may enhance its antioxidant properties, specifically against DPPH radicals. In the ABTS radical scavenging assay, both Q-40 and Q-60 demonstrated considerable antioxidant abilities.

The results were not quite the same as the studies of Hirose [5] and Li [11]. It may be due to the differences in the impurities in the extractions. The extraction method may contribute to the distinction as well.

The higher scavenging rates of Q-60 in both assays suggest that the extraction process, particularly the ethanol concentration, significantly influences the antioxidant potential of quinoa polysaccharides. This could be due to the selective extraction of specific polysaccharide fractions with higher antioxidant capacities. While both quinoa polysaccharide fractions show considerable antioxidant activity, their effectiveness is less than that of ascorbic acid. However, their natural origin and potential health benefits still make them attractive as antioxidant sources.

### 2.8. Anti-Proliferation Effect

**Figure 7:**
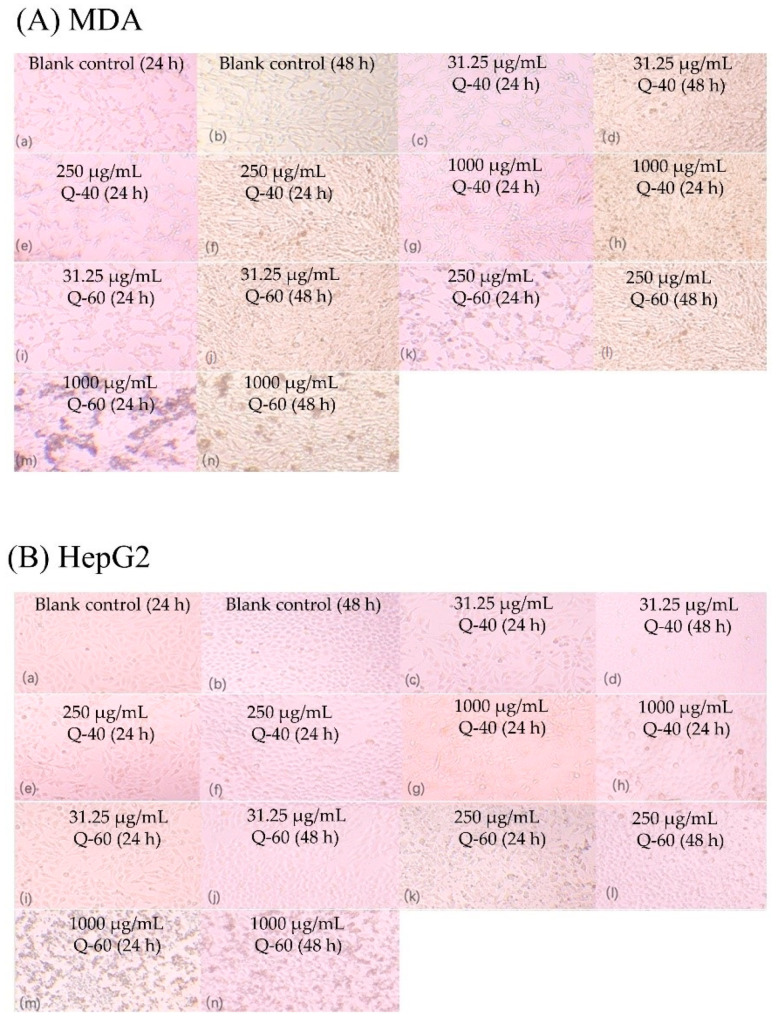
Effect of Q-40 and Q-60 on the proliferation of MDA and HepG2 cells. (**A**) Inhibition rate of proliferation of MDA-231 cells by Q-40 and Q-60; (**B**) inhibition rate of Q-40 and Q-60 on the proliferation of HepG2 cells.

Figure 7 presents the image of MDA and HepG2 cells after the treatment of Q-40 and Q-60 which indicated anti-proliferative activities of Q-40 and Q-60 against the two types of cells. For MDA cells cultivated for 24 h at lower concentrations (31.25 μg/mL), both Q-40 and Q-60 showed only a slight anti-proliferative effect (4.6%, 8.2%). Notably, for MDA cells cultivated for 48 h, Q-40 displayed a minor anti-proliferative effect at low concentrations (31.25 μg/mL), while Q-60 and Q-40 at other concentrations exhibited a pronounced promotion effect. Specifically, Q-60 showed the greatest proliferation-promoting effect at 1000 μg/mL, whereas the promotion effect of Q-40 increased with higher concentrations.

In HepG2 cells, both Q-40 and Q-60 demonstrated proliferation-promoting effects, and these effects increased with higher concentrations at 24 h. However, at 48 h, the promotional effects of both polysaccharides were not as evident, and the promotion effect was not apparent at concentrations of 250 μg/mL and higher.

In summary, the anti-proliferative activities of Q-40 and Q-60 varied depending on the concentration and cell type. While both polysaccharides exhibited anti-proliferative effects at lower concentrations and shorter incubation times in MDA cells, they demonstrated promotion effects at higher concentrations and longer incubation times. Conversely, in HepG2 cells, both polysaccharides displayed promotional effects at 24 h, but the effects were not significant at 48 h, particularly at higher concentrations.

The distinct patterns of cell proliferation induced by Q-40 and Q-60 raise questions about their mechanisms of action. It is possible that these polysaccharides interact with cell signaling pathways or cellular metabolism in a manner that varies over time and with concentration. The observed effects at different concentrations and time points could have implications for the therapeutic application of these polysaccharides. Understanding the optimal concentrations and exposure times will be crucial for maximizing their efficacy. Further research is needed to elucidate the molecular mechanisms underlying the observed effects, as well as to explore the impact of these polysaccharides on other cell types. Additionally, investigating the long-term effects of these compounds could provide deeper insights into their potential as therapeutic agents.

### 2.9. Anti-Microbial Ability

The antimicrobial abilities of Q-40 and Q-60 against *Candida albicans*, *Cryptococcus neoformans*, *Trichophyton rubrum*, *T. mentagrophytes*, *MRSA*, and *Escherichia coli* were assessed using the microdilution method, and the results are summarized in Table 3. Q-40 displayed inhibitory activity against methicillin-resistant *Staphylococcus aureus* and *Escherichia coli*, which are bacteria, with inhibition rates of 16.61 ± 0.87% and 3.39 ± 1.91%, respectively. However, no inhibitory effect of Q-40 was observed on *Candida albicans*, *Cryptococcus neoformans*, *Trichophyton rubrum*, and *T. mentagrophytes*, which are fungi. On the other hand, Q-60 exhibited inhibitory activity against *MRSA*, with an inhibition rate of 23.97 ± 0.64%. Similar to Q-40, Q-60 did not show any inhibitory effect on *Candida albicans*, *Cryptococcus neoformans*, *Escherichia coli*, *Trichophyton rubrum*, or *T*. *mentagrophytes.*

In summary, both Q-40 and Q-60 demonstrated some degree of antimicrobial activity against *MRSA*, while Q-40 also exhibited a minimal inhibitory effect on *Escherichia coli*. However, neither polysaccharide had any inhibitory effect on the other tested microorganisms, including *Candida albicans*, *Cryptococcus neoformans*, *Trichophyton rubrum*, and *T. mentagrophytes*.

## 3. Materials and Methods

### 3.1. Materials and Reagents

Quinoa was purchased from Shanxi Jiaqi Quinoa Development Co., Ltd. (Taiyuan, China). Ethanol, sulfuric acid, and trichloromethane were purchased from Guangzhou Chemical Reagent Factory (Guangzhou, China). Petroleum ether and phenol were purchased from Tianjin Damao Chemical Reagent Factory (Tianjin, China). Normal butanol was purchased from Tianjin Yongda Chemical Reagent Co., Ltd. (Tianjin, China). Monosaccharide standards, including rhamnose, xylose, glucose, etc., were chromatographically pure and purchased from Shanghai Yuanye Biotechnology Co., Ltd. (Shanghai, China). KCZ, penicillin, and TCS was purchased from Guangdong Jianyang Biotechnology Co., Ltd. (Guangzhou, China).

### 3.2. Extraction of Polysaccharides

A total of 1 kg of quinoa was initially washed with deionized water, crushed, and then passed through a 60-mesh sieve. Next, petroleum ether was added, and reflux extraction was conducted for 1 h each time. The mixture was then filtered, and the residue was subjected to two cycles of reflux extraction with 95% ethanol for 2 h each, aiming to remove pigments, lipids, and small molecules such as monosaccharides. Subsequently, the resulting filter residue was dried in an oven at 45 °C for 48 h, yielding the quinoa sample powder.

For the pretreated quinoa sample, it was mixed with deionized water at a ratio of 1:20 (g/mL) and subjected to reflux in a water bath at 100 °C for 2 h. Afterward, the sample underwent centrifugation at 4500 rpm for 15 min, and the supernatant was collected and concentrated under reduced pressure. The process was repeated once. To remove the residual Sevag reagent, a Sevag reagent (chloroform: *n*-butanol = 4:1) treatment was performed, and this operation was repeated 4–5 times. The remaining Sevag reagent was then removed by spin evaporation under reduced pressure at 50 °C. After deproteinization, the quinoa polysaccharide was prepared as a 10 mg/mL polysaccharide solution and mixed with anhydrous ethanol to achieve a 40% ethanol concentration. The resulting mixture was refrigerated overnight at 4 °C, and the precipitate was collected by centrifugation (4500 rpm) for 15 min. This precipitate was later re-dissolved in deionized water and dialyzed in dialysis bags (M_W_ 3500 Da) for 48 h. During this period, the water was changed every 16 h. The polysaccharide solution in the dialysis bags was collected and freeze-dried, resulting in the 40% fraction of polysaccharide Q-40. The same method was employed to obtain Q-60 with the ethanol concentration being increased to 60%.

### 3.3. Determination of Physical and Chemical Properties

The determination of polysaccharide content was conducted through the sulfuric acid–phenol method. Briefly, accurately weigh 20 mg of standard glucose and dissolve it in a 500 mL volumetric flask, filling up to the mark with water. Aliquot 0.4, 0.6, 0.8, 1.0, 1.2, 1.4, 1.6, and 1.8 mL of this solution, each diluted with distilled water to a final volume of 2.0 mL. Subsequently, add 1.0 mL of 6% phenol and 5.0 mL of concentrated sulfuric acid to each aliquot. Mix thoroughly and allow to stand for 30 min. Measure the absorbance at 490 nm. Use 2.0 mL of water treated with the same coloring agents as a blank. Plot a standard curve with polysaccharide microgram amounts on the x-axis and optical density values on the y-axis. The standard curve regression equation for the determination of polysaccharide content was y = 0.0061x − 0.0058, with a high correlation coefficient of R^2^ = 0.9981. Here, the mass concentration of D-glucose served as the horizontal coordinate, and the absorbance was the vertical coordinate [24].

The determination of protein content was conducted through the Kormas Brilliant Blue method. Briefly, dissolve 100 mg of Coomassie Brilliant Blue G-250 in 50 mL of 95% ethanol, then add 100 mL of 85% phosphoric acid, and complete the volume to 200 mL with distilled water. Use antibodies as the standard protein to construct a standard curve in the range of 20 µg to 150 µg per 100 µL. Dissolve the sample to be analyzed in PBS solution. Dilute the concentrated dye-binding solution with distilled water at a ratio of 1:4. Should any precipitation form, filter the solution to remove the precipitate. Add 5 mL of the diluted dye-binding solution to each sample and incubate for 5 to 30 min. After the dye binds with the protein, the color will change from red to blue. Measure the absorbance at a wavelength of 595 nm. Note that the color development reaction should not exceed 30 min.

The standard curve regression equation was y = 0.005x + 0.3556, with a strong correlation coefficient of R^2^ = 0.9982. The mass concentration of bovine serum albumin was used as the horizontal coordinate and the absorbance as the vertical coordinate [25].

### 3.4. Rheological Characteristics Analysis

Rheological measurements were carried out using a rheometer (RST-CPS, Brookfield, WI, USA) equipped with a 40 mm parallel plate. The linear viscoelastic properties, including the storage modulus (G′) and loss modulus (G″), of both Q-40 and Q-60 at various concentrations were determined using dynamic strain sweep and dynamic frequency sweep techniques at room temperature (25 °C).

To perform the measurements, the samples were dissolved at concentrations of 5, 10, 20, and 40 mg/mL in deionized water. The dynamic frequency sweep was conducted in the frequency range from 0.1 to 100 Hz, with 1 mL of the sample injected for each measurement.

### 3.5. Monosaccharide Composition Analysis

The determination of monosaccharide composition was carried out using ion chromatography [26]. A ten-milligram sample in an ampoule was mixed with 10 mL of 3 mol/L trifluoroacetic acid (TFA) and then hydrolyzed at 120 °C for 3 h. The acid hydrolysis solution was accurately transferred to a tube, dried with nitrogen, and then 5 mL of water was added and mixed thoroughly using vortexing. Next, 100 μL of the solution was aspirated and combined with 900 μL of deionized water. After centrifugation at 12,000 rpm for 5 min, the supernatant was collected for analysis using ion chromatography by Thermo Scientific Dionex, Waltham, MA, USA. For the chromatography, the following conditions were applied: The chromatographic column used was DionexCarbopacTMPA20 (3 × 150). The mobile phase comprised three components: A: H_2_O; B: 15 mM NaOH; C: 15 mM NaOH and 100 mM NaOAc. The flow rate was set to 0.3 mL/min, and the injection volume was 5 µL. The column temperature was maintained at 30 °C, and detection was performed using an electrochemical detector.

To create the monosaccharide standards, 16 monosaccharides (fucose, rhamnose, arabinose, galactose, glucose, xylose, mannose, fructose, ribose, galacturonic acid, glucuronic acid, aminogalactose hydrochloride, glucosamine hydrochloride, N-acetyl-D glucosamine, gulo-glucuronic acid, and mannuronic acid) were added to a 10 mg/mL standard solution. Each monosaccharide standard solution was precisely prepared to a concentration of 5 mg/L. The monosaccharide concentrations were determined based on the monostandard method, and the molar ratios were calculated using the respective molar masses of the monosaccharides.

### 3.6. Molecular Weight

High-Performance Gel Permeation Chromatography (HPGPC) was used to analyze polysaccharide molecular weight and purity [27]. The samples and standards were weighed precisely, and the samples were prepared at 5 mg/mL and centrifuged at 12,000 rpm for 10 min. And then, the supernatant was filtered through a 0.22 μm microporous membrane. The samples were transferred into 1.8 mL injection vials. Chromatography conditions: Chromatographic column: BRT105-104-102 tandem gel column (8 × 300 mm, fractionation range, 5000–300,000). Mobile phase: 0.05 mol/L NaCl solution. Flow rate: 0.6 mL/min, column temperature: 40 °C. Injection volume: 20 μL. Detector: RI-10A differential detector.

### 3.7. SEM Analysis

A small amount of Q-40 and Q-60 polysaccharides were weighed onto the sample stage, sprayed with gold, and placed under the scanning electron microscope for observation and filming. The morphology was obtained after the instrument was set up and stabilized.

### 3.8. Thermal Stability Analysis

The thermal stability of polysaccharides was determined using the following methods [28]. Appropriate amounts of Q-40 and Q-60 polysaccharides were weighed onto the sample bench, warmed up to 600 °C at a rate of 10 °C/min, and analyzed by thermogravimetry (TG), differential thermal gravity (DTG), and differential scanning calorimetry (DSC). The temperature and weight loss curves were used as the X and Y axes to plot the thermogravimetric variation curves.

### 3.9. Antioxidant Activity Analysis

#### 3.9.1. DPPH Radical Scavenging Activity

The determination of DPPH radical scavenging activity was conducted with slight modifications [29]. The DPPH (2,2-diphenyl-1-picrylhydrazyl) assay is based on the reduction of the DPPH radical, a stable free radical with a characteristic absorbance at 517 nm. Antioxidants in the test sample can donate hydrogen to DPPH, causing it to lose its radical character and resulting in a decrease in absorbance. Polysaccharide solutions were prepared at concentrations of 0, 1.0, 2.0, 3.0, 4.0, and 5.0 mg/mL, and their respective DPPH radical scavenging rates were measured. The absorbance values at 517 nm were recorded using a spectrophotometer, and the DPPH radical scavenging ability was calculated using ascorbic acid as the control group with the following formula:

Scavenging rate/% = (1 − (A1 − A2)/A) × 100%(1)

where A1 represents the absorption of the DPPH solution with the polysaccharide sample, A2 is the absorption of anhydrous ethanol with the polysaccharide sample, and A is the absorption of the DPPH solution with deionized water.

#### 3.9.2. ABTS Radical Scavenging Activity Analysis

The determination of ABTS radical scavenging activity was conducted following a method reported by Meng with slight modifications [30]. The ABTS (2,2′-azino-bis(3-ethylbenzothiazoline-6-sulfonic acid)) assay involves the generation of the blue-green ABTS+ radical cation through the reaction of ABTS with a suitable oxidizing agent (like potassium persulfate). Antioxidants in the test sample can quench the ABTS•+ radical cation, leading to a decrease in absorbance at 734 nm. Polysaccharide solutions were prepared at concentrations of 0, 1.0, 2.0, 3.0, 4.0, and 5.0 mg/mL, and their respective ABTS radical scavenging rates were determined. The solution was filtered through a 0.45 μm microporous membrane and then diluted with phosphate-balanced solution (PBS, 5.0 mmol/L, pH 7.4) to achieve an absorbance value of 0.70 ± 0.02 at 734 nm before measurement. Next, 0.4 mL of the sample solution with different concentrations was added to 4 mL of the working solution, shaken vigorously for 30 s, and left to stand for 6 min in the dark. The absorbance was then measured at the same wavelength. The scavenging ability of ABTS radicals was calculated as follows:

Scavenging rate/% = (1 − (A1 − A2)/A) × 100%(2)

where A1 represents the ABTS solution with the polysaccharide sample solution added, A2 is the ABTS solution with deionized water and the polysaccharide sample solution added, and A is the ABTS solution with deionized water added.

### 3.10. Anti-Proliferation Activity Analysis

The Cell Counting Kit-8 (CCK-8) assay was employed to evaluate the anti-proliferation effect of quinoa polysaccharides on HepG2 and MDA cells [30,31]. During the logarithmic growth phase, HepG2 and MDA cells were seeded into 96-well plates at a density of 5000 cells/well and then placed in a thermostatic incubator at 37 °C with 5% CO_2_ overnight. Subsequently, the supernatant medium in the wells was discarded, and the cells were washed twice with sterile PBS. Next, a new DMEM medium solution containing various concentrations of quinoa polysaccharide extracts (ranging from 0.03125 to 1.00 mg/mL) was added to the wells. After 24 h and 48 h of incubation, the cells were removed, photographed, and then incubated with the Cell Counting Kit-8 working solution for 1 to 2 h. The absorbance at 450 nm was measured using a microplate reader to determine the inhibition rate of cell proliferation. The calculation of the inhibition rate is as follows:

Inhibition rate = (1 − A1/A) × 100%(3)

where A1 represents the absorbance of the experimental group (cells treated with quinoa polysaccharide extracts), and A is the absorbance of the control group (cells without quinoa polysaccharide treatment).

### 3.11. Anti-Microbial Activity

To evaluate the antifungal activity against filamentous fungi, *Trichophyton rubrum* and *T. mentagrophytes* were individually cultured for 2 weeks at 28 °C on SDA (Sabouraud Dextrose Agar) to produce conidia. A mixed suspension of conidia and hyphae fragments was obtained by covering the fungal colonies with sterile saline (0.85%) and gently rubbing the colonies with an inoculation loop. The resulting suspension was then filtered through four layers of sterile lens paper to remove the hyphae, and the remaining conidia were collected by centrifugation at 1000× *g* for 10 min. The conidia were washed twice with sterile saline, and their concentration was adjusted to 1 × 10^4^ cells/mL using a hemocytometer for cell counting.

Antifungal susceptibility testing was conducted following the guidelines outlined in documents M38-A2 and previous research, with minor modifications [32,33]. The testing medium used was RPMI 1640 with L-glutamine buffered to pH 7.0 with 0.165 M 3-(N-morpholino) propanesulfonic acid (MOPS) and supplemented with 2% glucose (*m*/*v*). For the assay, 195 μL of the prepared conidia or spore suspension was seeded into 96-well plates, and each well was supplemented with 5 μL of the tested agents. Three replicates were used for each treatment, with ketoconazole serving as a positive control. The 96-well plates were then incubated at 28 ± 2 °C for 7 days, and the optical density was measured at 510 nm using a microplate reader.

For antifungal susceptibility testing of yeasts, a micro-broth dilution assay was employed. The test compounds were dissolved in DMSO at a stock concentration of 4 mg/mL and stored at 4 °C for the bioassays. Exponentially growing cultures of each strain were prepared from overnight cultures and adjusted to an OD (optical density) value of approximately 0.5 at 600 nm. The cultures were then diluted 1:1000 in broth (*Cryptococcus neoformans* was directly used at OD600 (optical density at 600 nm) = 0.5) and added to a 96-well plate at a volume of 195 µL per well. Ketoconazole (at a concentration of 10 µg/mL) was used as a positive control for *Candida albicans* and *Cryptococcus neoformans*. After incubation for 48 h, the plates were read at 600 nm, and inhibition was calculated by subtracting the absorbance of the blank wells, dividing by the average value for the DMSO-only wells, and multiplying by 100.

Similarly, samples were tested for planktonic microbial growth inhibition using the micro-broth dilution assay mentioned above [34]. The compounds and standard drugs were prepared in DMSO. Exponentially growing cultures of *Staphylococcus aureus*, *Escherichia coli*, *Streptococcus mutans*, and *Streptococcus sobrinus* were prepared from overnight cultures and adjusted to an OD value of 0.5 at 600 nm. The cultures were then diluted 1:1000 in broth and added to a 96-well plate at a volume of 195 µL per well. Penicillin G and tetracycline (at a concentration of 10 µg/mL in DMSO) were used as standard drugs. After incubation for 24 h, the plates were read at 600 nm, and inhibition was calculated using the following formula:

Inhibition Rate = (1 − (A1 − A2)/A) × 100%(4)

where A1 stands for the absorbance of the experimental group, A2 is for the blank group, and A is for the control group.

### 3.12. Data Analysis

Each experiment was replicated three times, and the mean was used. Data analysis was performed using Excel 2010. Origin 8.6 was used to plot the graphs. The data are expressed as the mean ± standard deviation (mean ± SD).

## 4. Conclusions

The current study focused on the extraction of two polysaccharides from quinoa using the ethanol grading method, namely Q-40 and Q-60. The analysis revealed that Q-40 had a polysaccharide content of 58.29% and a protein content of 8.27%, while Q-60 had a polysaccharide content of 62.15% and a protein content of 9.6%. Both Q-40 and Q-60 displayed heterogeneous polysaccharide profiles, with Q-40 showing three molecular weight peaks and Q-60 exhibiting two peaks. Moreover, both polysaccharides exhibited significant DPPH and ABTS radical scavenging abilities, indicating potent antioxidant properties.

The antioxidant ability of polysaccharides is crucial for combating oxidative stress, which plays a role in aging and various human diseases, such as diabetes and liver damage. Q-40 and Q-60 exhibited considerable antioxidant activity, particularly Q-60, suggesting their potential application as natural antioxidants in the nutritional and pharmaceutical industries.

However, we have found that Q-40 and Q-60 exhibit almost equal percentages of polysaccharides (58.29% and 62.15%) but differ greatly in antioxidant activity, SEM image, and antimicrobial activity. This may be due to the structural variations in these polysaccharides. For instance, molecular weight, degree of branching, and the presence of different functional groups. Furthermore, the presence of other bioactive compounds along with their intermolecular interactions may have a great influence as well.

Regarding antimicrobial activity, Q-40 demonstrated antibacterial effects against methicillin-resistant *Staphylococcus aureus* and *Escherichia coli*, while Q-60 exhibited antibacterial effects against *methicillin-resistant Staphylococcus aureus*. Although the inhibition abilities were weaker than those of the positive control group, this finding aligns with observations from other plant polysaccharides. The exact mechanisms underlying the antimicrobial ability of quinoa polysaccharides require further investigation, considering that the polysaccharides are not 100% pure.

As for anti-proliferative effects, both Q-40 and Q-60 displayed inhibitory effects at low concentrations against MDA and HepG2 cells in in vitro experiments. However, no further effects on tumor cells were observed. In-depth in vivo studies are needed to explore the potential antitumor activity of quinoa polysaccharides. Moreover, our preliminary data indicated that Q-40 and Q-60 exhibit antioxidant, antimicrobial, and anti-proliferative activities. However, the mechanisms underlying how quinoa polysaccharides exert protective roles in aging processes and human disease development require further investigation.

In conclusion, this study highlights quinoa polysaccharides as promising candidates for food supplementation or products aimed at improving human health and well-being. The potent antioxidant, antimicrobial, and anti-proliferative activities of these polysaccharides hold significant potential for various applications, and further research is essential to fully understand their mechanisms and explore their therapeutic potential.

## Figures and Tables

**Table 1:** HPLC analysis of Q-40 and Q-60.

	Q-40			Q-60			
Monosaccharide	Ara	Glc	GalA	Ara	Gal	Glc	GalA
Molar Ratio	0.006	0.967	0.027	0.036	0.034	0.889	0.020

**Table 2:** Relative molecular masses of Q-40 and Q-60.

Sample	Q-40			Q-60	
	Peak 1	Peak 2	Peak 3	Peak 1	Peak 2
Mp	43,3062	155,898	38,510	38,739	8924
Mw	626,488	210,818	47,484	47,990	10,025
Mn	350,187	127,526	32,003	32,427	7601
PDI (Mw/Mn)	1.789	1.653	1.484	1.480	1.319

**Table 3:** Antimicrobial properties of Q-40 and Q-60. Ketoconazole (KCZ), used as a reference antifungal agent, and Penicillin and Tetracyclines (TCS), used as reference antibacterial agents, for comparative purposes.

Fungi					Bacteria	
	*Candida albicans*	*Cryptococcus neoformans*	*Trichophyton rubrum*	*T. mentagrophytes*	*MRSA*	*Escherichia coli*
Q-40	—	—	—	—	16.61 ± 0.87%	3.39 ± 1.91%
Q-60	—	—	—	—	23.97 ± 0.64%	—
KCZ (Ketoconazole)	89.82 ± 2.34%	100 ± 0.29%	98.17 ± 0.15%	—		
Penicillin				96.64 ± 0.21%	98.10 ± 0.25%	
TCS (Tetracyclines)						95.02 ± 1.08%

## Data Availability

The data presented in this study are available in article.
